# Safety profile, antiviral capacity, and liver protection of a nasal therapeutic vaccine in patients with chronic hepatitis B: Five-year-follow-up outcomes after the end of treatment

**DOI:** 10.3389/fmed.2023.1032531

**Published:** 2023-02-08

**Authors:** Mamun Al Mahtab, Sheikh Mohammad Fazle Akbar, Julio Cesar Aguilar, Osamu Yoshida, Sakirul Khan, Guillen Nieto Gerardo, Yoichi Hiasa

**Affiliations:** ^1^Department of Hepatology, Interventional Hepatology Division, Bangabandhu Sheikh Mujib Medical University, BSMMU, Dhaka, Bangladesh; ^2^Department of Gastroenterology and Metabology, Ehime University Graduate School of Medicine, Toon, Japan; ^3^Center for Genetic Engineering and Biotechnology, Havana, Cuba; ^4^Department of Microbiology, Faculty of Medicine, Oita University, Oita, Japan

**Keywords:** chronic hepatitis B, NASVAC, immune therapy, nasal therapeutic vaccine, 5-year follow-up

## Abstract

**Introduction:**

There is a pressing need to develop novel drugs for treating patients with chronic hepatitis B (CHB), as commercially available antiviral drugs are endowed with safety and efficacy concerns.

**Methods:**

A phase III clinical trial was conducted with a therapeutic vaccine containing two antigens of the hepatitis B virus (HBV; named NASVAC) in 78 patients with CHB expressing both HBV DNA and elevated levels of alanine aminotransferase (ALT) in the blood. Five years after the end of treatment (EOT), 60 NASVAC-recipient patients were enrolled in this long-term follow-up study to evaluate the safety, antiviral potential, and liver-protective capacity of NASVAC.

**Results:**

NASVAC exhibited an excellent safety profile 5 years after EOT. The levels of HBV DNA in the sera were reduced in 55 of the 60 patients, and 45 of them were negative for HBV DNA in the sera. ALT levels were also normalized in 40 of the 60 patients 5 years after EOT. None of the patients receiving NASVAC developed liver cirrhosis or cancer.

**Discussion:**

The present study is the first to exhibit long-term follow-up data of a finite immune therapy for CHB that is safe and endowed with potent antiviral and liver-protecting capacities.

## Introduction

Hepatitis B virus (HBV) infection is a complex global public health problem. According to the World Health Organization (WHO), approximately 2 billion people (of the existing total world population of 7.6 billion) have acquired HBV infection at some point. Of these 2 billion people, an estimated 296 million are chronically infected with HBV, and hence, these people represent living and permanent reservoirs of HBV. This condition indicates that these patients have been expressing HBV DNA in their blood and that they would transmit HBV to normal and HBV-non-infected persons. Epidemiological data sponsored by the WHO indicate that approximately 12–25% of chronic HBV-infected subjects would develop evidence of liver damage in addition to HBV replication [a condition called chronic hepatitis B (CHB)] and progress to the development of complications, such as liver cirrhosis (LC) and hepatocellular carcinoma (HCC). In 2019, an estimated 882,000 people died of HBV-related complications (1).

Hepatitis B virus-related LC and HCC develop in patients with CHB after prolonged suffering, with exacerbation and remission of hepatitis, development of liver fibrosis, and malignant transformation of hepatocytes (2, 3). However, once LC or HCC is diagnosed, there is no curative therapy for these complications, and the quality of life is highly compromised with evidence of ascites, bleeding of esophageal varices, and several other LC-related symptoms. This imparts a severe burden on patients and the healthcare delivery systems of all countries. However, if patients with CHB can be appropriately treated and managed, complications such as LC and HCC may be controlled, and the number of HBV-related deaths can be reduced.

This frustrating picture of CHB-related complications and deaths has prevailed, despite the availability of potent antiviral drugs against HBV. Currently, two groups of medications are used to treat patients with CHB. These drugs include interferons (IFNs; standard and its pegylated forms) and nucleoside analogues (NUCs) (4, 5). The safety issues of IFNs, high cost, parental route of administration, and limited efficacy could not popularize IFNs as treatment modalities for CHB, even in developed and prosperous countries (6, 7). With the introduction of NUCs for the treatment of CHB, considerable optimism was initiated among both patients and physicians. NUCs can be taken orally and can induce HBV reduction or even negativity of HBV DNA in many patients with CHB (8, 9). Approximately 25 years have passed since the entry of NUCs for the treatment of CHB (10–12). It is now apparent that NUCs cannot be the treatment of choice for CHB due to the following reasons: (1) NUCs cannot eradicate all forms of HBV DNA, especially closed covalently circular DNA (cccDNA), which acts as a template for new DNA formation; (2) NUCs therapy must be used for several years or even for a patient’s whole life. Additionally, stopping NUCs usually induces the reappearance of HBV replication and a hepatic flare that may be life-threatening or even fatal; (3) the efficacy of NUCs to contain progression to LC and HCC is not satisfactory; and (4) the immunomodulatory capacity of NUCs is insignificant. Thus, NUCs exhibit only antiviral potential, and their ability to induce sustained control of liver damage is negligible (13).

Ideally, the most effective way to treat patients with CHB is to develop a new drug for CHB, which is safe, allow a finite duration of usage, and able to control the HBV replication and induce sustained control of liver damage. To develop a novel therapeutic approach, we critically analyzed the mechanisms of CHB pathogenicity and the advantages and disadvantages of all available anti-HBV drugs. HBV is a non-cytopathic virus, and aberrant host immunity is mainly responsible for inducing HBV-related liver damage, persistence HBV replication, and development of HBV-related complications (14, 15). This has opened a new field of development for immune therapeutic agents for treating patients with CHB. The development of immune therapy against CHB began in the mid-1990s (16, 17). However, the following factors seem to be responsible for the lack of a recognized and effective therapeutic agent for CHB (17). In most instances, preclinical animal studies have not retrieved proper evidence to translate immunotherapy in patients with CHB. In addition, most immune therapeutic approaches have been used in pilot or observational studies. Thus, they exhibit encouraging outcomes at the end of treatment (EOT). However, these studies were not designed to provide long-term follow-up data to assess the therapeutic implications of these agents. Finally, there is little or no information regarding the mechanism of action of immune therapeutics (17).

After analyzing these limitations, we developed a new mode of immune therapeutic agents in the early 2000s by combining two HBV antigens: hepatitis B surface antigen (HBsAg) and hepatitis B core antigen (HBcAg; named NASVAC). The antigen-specific immune therapeutic models were adapted as HBV-specific immunity is endowed with antiviral and liver-protective capacities. NASVAC was optimized for use *via* the nasal route so that it can be used easily by patients in developing and resource-constrained countries (80% of patients with CHB reside in these countries) (18). The development of NASVAC has progressed systematically. The safety and efficacy of NASVAC and its mechanism of action have been systematically evaluated in normal mice and human volunteers (19). This was followed by studies of NASVAC in HBV transgenic mice (HBVTM) that expressed HBV DNA and HBsAg in the sera. The study mainly evaluated the mechanism underlying the therapeutic efficacy of NASVAC in HBVTM (20). We subsequently moved from the benches to the bedsides. A phase I/II clinical trial of NASVAC was performed in patients with CHB expressing HBV DNA and with liver damage (21). This was followed by a phase III clinical trial of NASVAC in patients with CHB. NASVAC showed profound safety, potent antiviral effects, and considerable liver-protecting potential compared with other commercially available antiviral drugs at the end of treatment (EOT) (22). To make the entire trial with NASVAC in CHB an evidence-based trial, we published the safety and efficacy profiles of NASVAC 24 weeks (22), 2 years (23), and 3 years (24) after EOT.

The present communication provides information on the safety, antiviral potential, and liver-protecting capacity of NASVAC 5 years after EOT. As NASVAC is a finite mode of therapy consisting of only 20 weeks, this study has a direct implication for “Control of Hepatitis by 2030.”

## Materials and methods

### Ethics statement

The study was approved by Bangabandhu Sheikh Mujib Medical University, Dhaka, Bangladesh, with project identification code No. BSMMU/2010/2363, dated March 1, 2010. The study was registered in ClincalTrials.Gov, with identification No. NCT10374308 (22). All patients provided written consent prior to the study, and the procedures were followed in accordance with the ethical guidelines of the Declaration of Helsinki (1964, amended recently in 2008).

### A comprehensive description of phase III clinical trial with NASVAC in patients with CHB

The present study has been planned to provide information on the 5-year follow-up data of NASVAC-treated patients with CHB, and a short and comprehensive description of phase III clinical trials of NASVAC is provided. NASVAC was prepared with a 1:1 formulation of 100 μg HBsAg (Pichia pastoris-derived recombinant HBsAg subtype adw2) and 100 μg HBcAg (purified *Escherichia coli*-expressed recombinant full-length HBcAg). The entire phase III clinical trial was conducted at the Bangabandhu Sheikh Mujib Medical University, Dhaka, Bangladesh, and Farabi Hospital, Dhaka, Bangladesh. A total of 78 patients with CHB participated in a phase III trial of NASVAC.

The diagnoses of patients enrolled in this study were based on serological, biochemical, virological, and imaging assessments. All the patients were diagnosed with HBV infection. The patients were treatment-naïve and did not consume any medications with direct antiviral properties against HBV. Patients of both sexes were enrolled in the study. All of them were positive for HBsAg for more than 6 months and were positive for HBV DNA in the sera. The alanine aminotransferase (ALT) levels at enrolment were above the upper limit of normal (ULN). HBV DNA levels were > 10^3^ copies/mL for hepatitis B e antigen [HBeAg (−)] and 10^4^ for HBeAg (+) patients.

The exclusion criteria included all patients with CHB in the immune tolerant and inactive HBV carrier state with normal ALT levels. All patients were free of LC and HCC development. Patients who were positive for hepatitis C virus, hepatitis delta, or human immunodeficiency virus were excluded from the study. In addition, patients receiving CHB treatment were not enrolled. All types of critically ill patients, patients with hypertension, hyperthyroidism, epilepsy, malignancies, or any non-controlled systemic disease, pregnant or nursing women, and women of fertile age without any contraceptive methods were excluded from the study. Patients with known severe allergic conditions or hypersensitivity, severe psychiatric dysfunction, or another limitation that prevented their consent were not included in the trial. Patients with autoimmune diseases or treatment with immunosuppressive or immune modulator drugs during or in the 6 months prior to the study, and patients with a history of alcohol or drug abuse were also excluded. Patients with other hepatic diseases of different etiologies and very high levels of ALT at the beginning of treatment (ALT > 500 U/l) suggest unstable disease or acute flares over ten times the ULN.

In this follow-up, 5 years after EOT, 60 patients were properly evaluated for safety and efficacy. These patients physically participated in the hospital and completed a questionnaire as per the permission and recommendations of the institutional review board. Blood was collected from all patients to assess the safety and efficacy parameters. The patients were advised to remain abstained from any recommended and commercially available antiviral drugs, such as type-A interferon or nucleoside analogues, during their follow-up period. They were also asked not to consume any direct immune modulators such as cytokines, chemokines, and growth factors. Abdominal ultrasonography was performed to determine the occurrence of complications such as LC and HCC. Detailed assessments of the ear, nose, and throat (ENT) were performed by an ENT specialist, as NASVAC was administered intranasally. As all sorts of analyzes were possible in 60 patients, this could not be performed in additional 18 patients in a phase III trial who could not attend hospitals physically because of variable circumstances. We contacted these 18 patients *via* phone. The principal investigator (Mamun AL Mahtab) talked to them and asked about their well-being regarding safety and clinical status.

### Profile of the patients participated physically in follow-up 5 years after EOT

The clinical profiles of 60 patients who physically attended the hospital for a 5-year follow-up after EOT are shown in Table 1. These data indicate their profile during study commencement. The age of the patient was 29.8 ± 7.00 years (range; 18–50 years). Of the patients, eight were women and 52 were men. The levels of HBV DNA before the start of NASVAC therapy ranged between 1.7 × 10^3^–1.0 × 10^13^ copies of HBV DNA/mL. All patients had a serum ALT level above the ULN (> 42 U/l) during enrollment in the phase III clinical trial (Table 1). The level of fibrosis was mild.

**Table 1:** Data of the 60 patients during enrollment in the phase III trial.

Variables	Values
Total numbers	60
Male: Female	52:8
Age (years)	29.8 ± 7.008
HBV DNA (copies/mL)	4.9 × 10^4^ (Range; 1.7 × 10^3^–1.0 × 10^13^)
Alanine aminotransferase (IU/L)	58.8 ± 31.5
Level of fibrosis	F0-F2

The values of different parameters are provided as absolute numbers (numbers of patients observed and male; female ratio), mean and standard deviation (age and ALT), or median and range (HBV DNA). The levels of fibrosis were mild.

### Quantification of safety parameters and efficacy in 60 patients who attended follow-up 5 years after EOT

A short account of the procedures is provided for ready reference. The general parameters of inflammation and serum levels of bilirubin, albumin, and creatinine were determined using the same methods used for the total duration of follow-up (15–17).

### Quantification of serum HBV DNA levels

Serum HBV DNA was quantified using a polymerase chain reaction method (Amplicon HBV Monitor Assay, RT-PCR; Roche Molecular Systems, CA, United States). The lower limit of detection was 250 copies of HBV DNA/mL.

### Assessment of ALT

Serum ALT levels were measured using a commercial kit approved by the hospital that provided permission from the institutional review board.

### Abdominal ultrasonography and upper abdominal endoscopy

Ultrasonography was performed by the same physician who performed abdominal ultrasonography at different points during follow-up (Pre-inclusion, EOT, 24 weeks, 2 years, and 3 years after EOT).

### Assessment of esophageal varices

Upper gastrointestinal endoscopy was performed in susceptible patients, and the principal investigator performed the procedure.

### Assessment of nasal complications (if Any) due to NASVAC administration

To assess complications due to the nasal administration of NASVAC, ENT specialists rigorously evaluated the patients.

## Results

### Assessment of the safety issues of NASVAC 5 years after the EOT

Nasal therapeutic vaccine (NASVAC) is a new therapeutic vaccine containing comparatively high levels of two antigens: a 1:1 formulation of 100 μg HBsAg (Pichia pastoris-derived recombinant HBsAg subtype adw2) and 100 μg of HBcAg (purified *E. coli*-expressed recombinant full-length HBcAg). Although manufactured under good manufacturing practice conditions, safety concerns have been a pertinent issue in its use in human beings. Sixty patients attended the 5-year follow-up at the hospital. However, safety issues were evaluated in an additional 18 patients with CHB enrolled in the initial phase III clinical trial *via* telephone. All patients were alive, and death of any patient within these 5–6 years period was not reported. None of the patients had any complaints regarding the safety of NASVAC. As NASVAC was administered *via* the nose, ENT specialists evaluated 60 physically attending patients to assess if there were any complications related to nasal administration of NASVAC. Most importantly, complications of NASVAC may occur in the liver and kidney. As shown in Table 2, the mean bilirubin, albumin, and creatinine levels were within the normal range in all patients receiving NASVAC 5-years after EOT. Ultrasonographic assessment of the abdomen also showed that none of the patients progressed to LC or HCC at 5-year after EOT. This suggests that NASVAC is one of the safest medications for CHB used till now.

**Table 2:** Parameters of liver and kidney function of 60 patients at a basal level, end of treatment (EOT) and 5-years after EOT.

Variables	Basal level	EOT	5-year after EOT
Bilirubin (mg/dl)	0.63 (0.2–1.26)	0.7 (0.4.1.16)	0.7 (0.48–1.8)
Albumin (gm/dl)	3.7 (3.4–4.2)	4.1 (3.6–4.6)	4.87 (3.99–5.83
Creatinine (mg/dl)	0.99 (0.46–1.8)	1.0 (0.62–1.42)	1.01 (0.5–1.31)

Basal level: the day of 1st administration; EOT: end of treatment with five nasal administration of nasal therapeutic vaccine (NASVAC), followed by five nasal and subcutaneous administration of NASVAC. Five years after EOT: 5 years after the end of therapy during long-term follow-up; Data are shown as median and range.

### Decrease of HBV DNA in the sera of NASVAC-treated patients with CHB 5 years after EOT

This study revealed data on the efficacy of NASVAC treatment 5 years after EOT. At the start of the phase III clinical trial, HBV DNA was detected in all the included patients (this was one of the essential inclusion criteria of the trial). The levels of HBV DNA varied considerably among patients on the day of the first immunization *via* the nasal route (basal level). All patients had a minimum level of 1.0 × 10^3^ copies of HBV DNA in their sera, and the maximum amount was 1.0 × 10^13^ copies/mL. Five year after EOT, HBV DNA was reduced in 55 of the 60 patients with CHB. Of these 55 patients, HBV DNA was undetectable in 45 patients 5 years after EOT (Table 3). This decline in HBV DNA by NASVAC represents the extraordinary long-term antiviral potential of NASVAC. HBV DNA negativity in a considerable number of patients represents the sustained effect of an immune therapeutic agent on HBV DNA.

**Table 3:** Sustained antiviral effect of NASVAC on serum HBV DNA 5-years after EOT.

Variables	Basal level (*N* = 78)	Five years after EOT (*N* = 60)
HBV DNA (copies/ml)	1.0 × 10^3^–1.0 × 10^13^	3.0×10^2^–3.5×10^5^
HBV DNA (positive)	78	15
HBV DNA (undetectable)	0	45
Highest levels of HBV DNA	1.0 × 10^13^	3.5×10^5^

#### Hepatitis B virus DNA in NASVAC-recipients CHB patients at basal point, and 5 years after EOT.

To have more insights about this, we analyzed HBV DNA at different time points following NASVAC therapy. HBV DNA were measured ten times within 5 years. The time periods are (1) Base line before administration of therapeutic vaccine, (2) 12 weeks after trial commencement, (3) 24 weeks after trial commencement, (4) 48 weeks, that means 24 weeks after end of treatment [EOT]), (5) 72 weeks (1 year after EOT, (6) 72 weeks after study commencement [1.5 year after EOT], (7) 120 weeks after study commencement, (8) 2 years after EOT, (9) 3 years after EOT and (10) 5 years after EOT.

**Figure 1:**
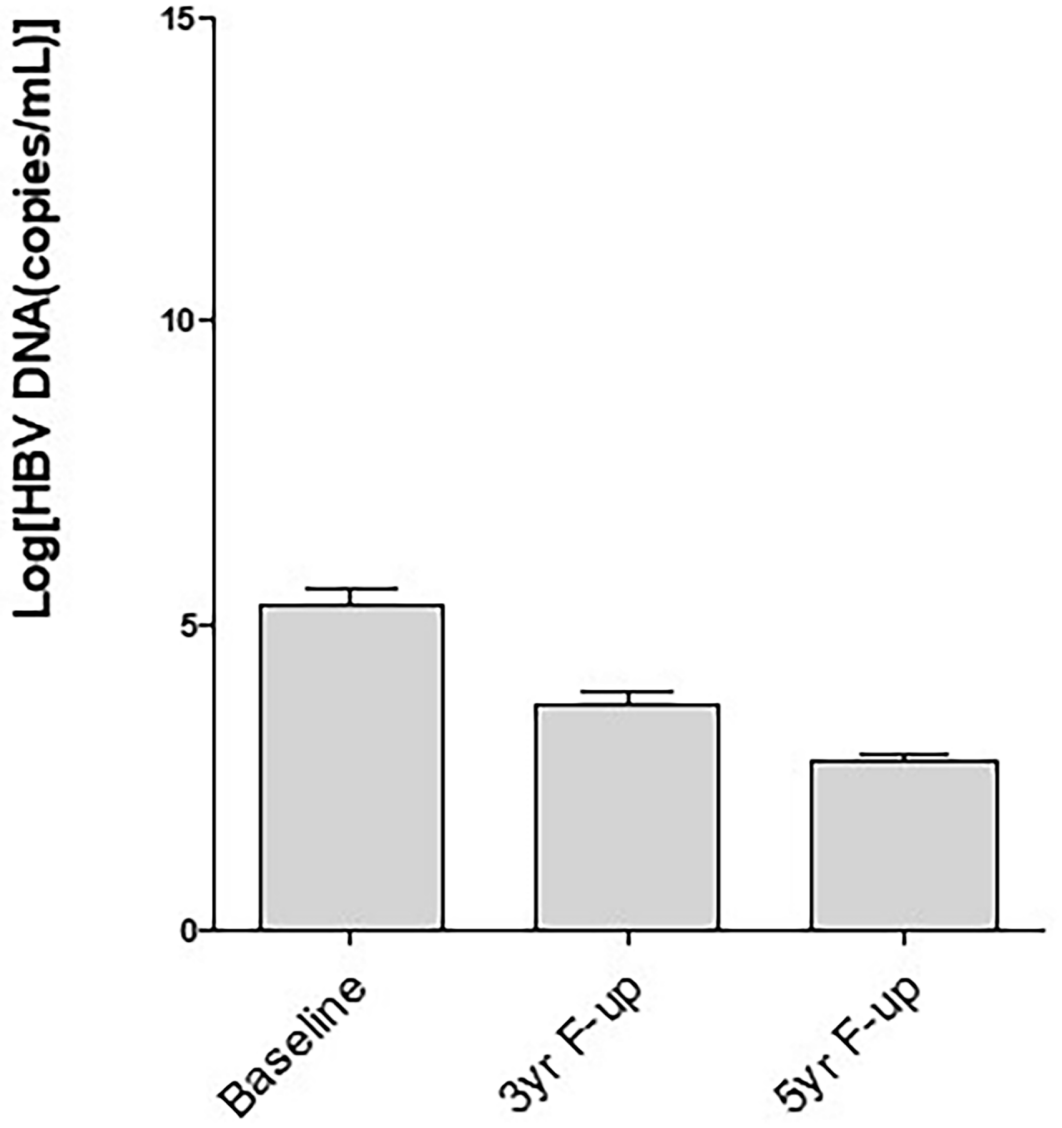
Decrease of hepatitis B virus (HBV) DNA in the sera along with time after therapy with a therapeutic vaccine (NASVAC). Baseline: The levels of HBV DNA evaluated before start of therapy; 3 yr-F-up: The level of HBV DNA 3-year after end of therapy; 5-yr-F-up: The levels of HBV DNA 5-year after end of treatment. Mean and standard deviation of HBV DNA of the evaluated patients have been shown.

The levels of HBV DNA exhibited considerable heterogenicity, however, HBV DNA decreased along with time and these have been published during follow up at 24-week after EOT (22), two-years (23) and 3 years after EOT (24). As shown in Figure 1, the mean levels of HBV DNA decreased further 5-years after EOT compared to their values of three-years after EOT. More analyzes showed that only 18 of 59 CHB patients were negative for HBV DNA in the sera 3-years after EOT (23). It is extremely promising that 45 of 60 patients in the present cohort became negative for HBV DNA in the sera, 5 year after EOT (Table 3).

**Figure 2:**
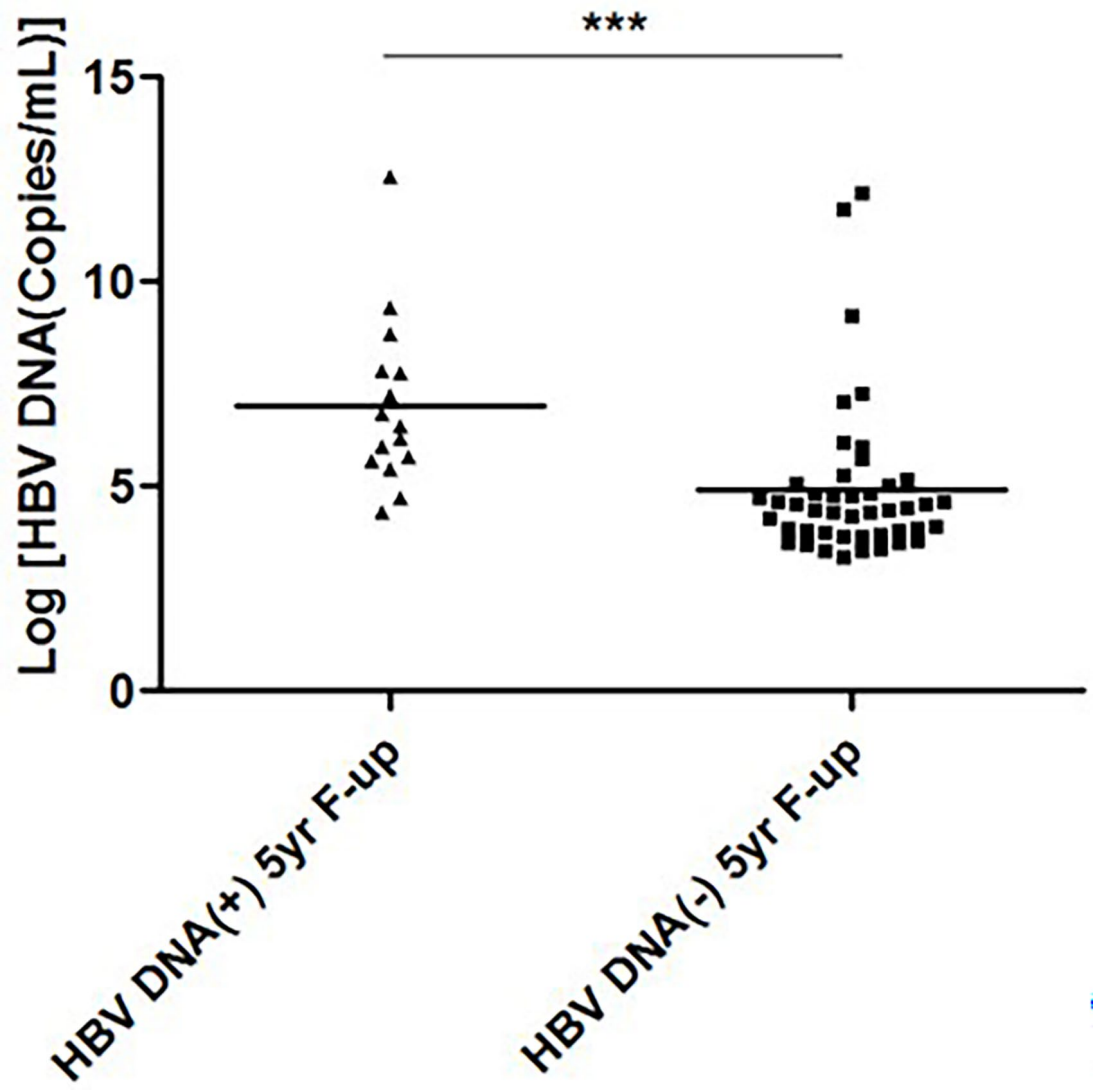
Hepatitis B virus (HBV) DNA levels at study commencement and HBV DNA-negativity 5-years after end of treatment. The levels of HBV DNA (log copies/ml) of two groups of patients at the time of study commencement has been cited. The levels of HBV DNA were lower in patients who became HBV DNA negative 5-years after end of treatment, compared to those who remain HBV DNA-positive 5-years after end of treatment.

Another important information was retrieved after analyzing the relation between levels of HBV DNA at study commencement and HBV DNA-negativity, 5-years after EOT. As shown in Figure 2, the levels of HBV DNA during study commencement were significantly lower in patients who became HBV DNA-negative (4.921 ± 1.520 log copies/ml; *p* < 0.001) compared to those who remain HBV DNA-positive (6.973 ± 2.088 log copies/ml; p < 0.001), 5 years after EOT.

#### hepatitis B e antigen-positivity and HBV DNA

**Figure 3:**
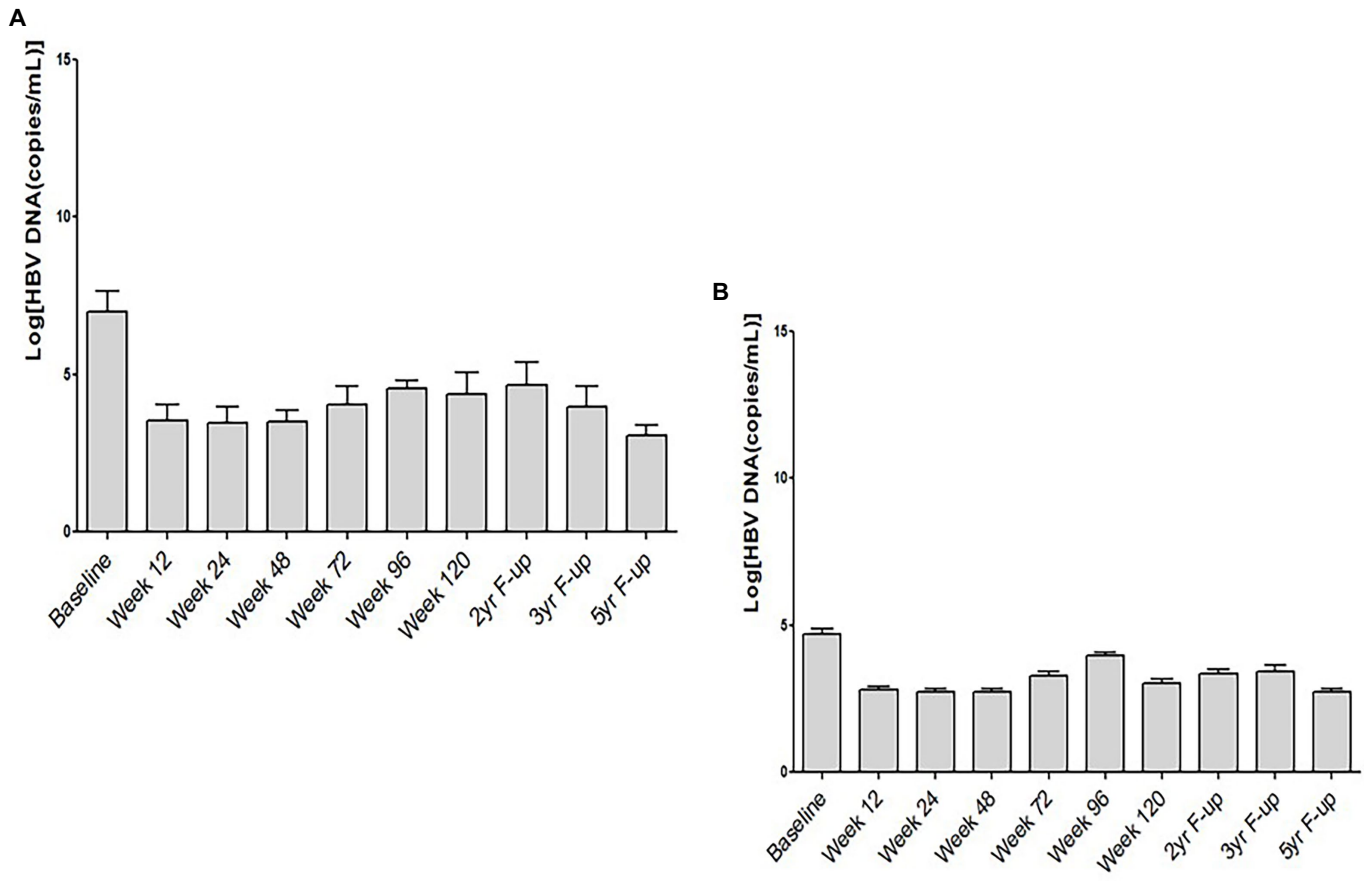
Kinetics of HBV DNA and hepatitis B e antigen (HBeAg) in chronic hepatitis B patients receiving therapeutic vaccine (NASVAC). The levels of HBV DNA at ten points after study commencement have been shown in HBeAg-positive **(A)** and HBeAg-negative **(B)** chronic hepatitis B (CHB) patients.

To have an understanding about the role of HBeAg-positivity in this clinical trial, we analyzed the HBV DNA levels in HBeAg-positive and HBeAg-negative CHB patients in this study. In this cohort of 60 patients, 12 patients were HBeAg-positive at the commencement of the clinical trial. The kinetics of HBV DNA of HBeAg-positive (Figure 3A) indicates that the levels of HBV DNA were considerably higher in these patients before study commencement. However, along with time, HBV DNA decreased in these patients (Figure 3A). The levels of HBV DNA was comparatively lower in HBeAg-negative patients before study commencement. These patients also exhibited reduction of HBV DNA along with time (Figure 3B) have been shown.

#### Normalization of ALT 5 years after EOT In patients with CHB receiving NASVAC

Patients with CHB had elevated levels of ALT (>ULN; >42 IU/l) when screened for inclusion in a phase III clinical trial. It is well known that ALT exhibits exacerbation and remission over the entire course of CHB. Although all 60 patients in this follow-up trial had elevated ALT levels at the start of therapy, a total of 40 patients with CHB had ALT within the ULN 5 years after EOT.

#### Hepatitis B e antigen negativity in two-thirds patients of CHB treated using NASVAC 5 years after EOT

There were 12 HBeAg-positive patients among the 60 patients in this cohort during the initial entry in the phase III trial. Of these 12 HBeAg-positive patients, eight became negative for HBeAg 5 years after EOT.

#### Development of anti-HBs in CHB patients due to NASVAC therapy and assessed 5 years after EOT

In this cohort, we enrolled 60 patients with CHB. It was seen that 20 patients developed anti-HBs in their sera. HBV DNA was not detectable in these patients. There was relation between levels of HBV DNA with anti-HBs positivity. The levels of HBV DNA in patients who became anti-HBs-positive were lower (4.957 ± 1.520 log copies/ml) than those in patients who were anti-HBs-negative (5.577 ± 2.340 log copies/ml), however, the levels did not reach statistical significance (*p* = 0.2326).

#### No progression to LC or/and HCC

The major problem with CHB is the development of complications such as LC and HCC, and these two morbidities are responsible for almost all HBV-related deaths globally. Thus, there was an urgent need to assess the progression of LC and HCC in patients receiving NASVACs. Patients with CHB receiving NASVAC were evaluated for the development of LC and HCC 5 years after EOT. The principal investigator recorded a history of illness of the study participants. Finally, if needed, patients underwent abdominal ultrasonography and upper gastrointestinal endoscopy. There was no evidence of LC development in any of the patients. Dilated esophageal varices were not found in any patient 5 years after EOT. Further, we did not notice incidence of HCC in any of the patients in this study.

## Discussion

In 2016, the WHO proposed the “Elimination of Hepatitis by 2030” concept after thoroughly discussing the situation at that time. Since then, new HBV infections have been reduced considerably due to various public health measures, including integrating hepatitis B vaccination into the Expanded Program of Immunization and reducing harm. However, these achievements would be short-lasting if proper treatment strategies are not adopted to treat millions of patients with chronic HBV-infected infection. This is because all chronic HBV-infected subjects are living and permanent reservoirs of HBV, transmitting HBV to healthy and HBV-uninfected persons (1). Another highly relevant finding indicates that only 10% of chronic HBV-infected subjects are aware of their infectious status. Several approaches have been adopted to discover millions of undetected chronic HBV-infected patients worldwide. Among these chronic HBV-infected subjects, an estimated 40–75 million patients with HBV DNA and liver damage (patients with CHB) deserve immediate and active therapy. Many patients with CHB would develop complications such as LC and HCC and follow extremely downhill courses clinically.

As of today, according to estimates of the WHO, only 5 million patients with CHB receive treatment. Six years have passed after the original declaration of “Elimination of Hepatitis by 2030”; however, the WHO aims to treat approximately 30–40 million patients by 2030. Based on the available information and experiences of the last four decades of treatment of patients with CHB by commercially available antiviral drugs such as IFNs and NUCs, it is clear that a vast number of patients with CHB cannot be treated by the available antiviral drugs due to their adverse effects, low efficacy, and infinite regimen of therapy, even if the missing patients with CHB can be brought to health services (1).

An ideal drug for CHB should also be used for a finite duration, with profound safety and efficacy. Immunotherapy has become a candidate for treating patients with CHB. However, there is no consensus among physicians and patients regarding the utility of this therapy. Immune treatment has been initiated in patients for four decades (9, 10). However, the nature of the drug, study design, lack of analysis of mechanisms, and appropriate follow-up did not allow immune therapy to stand the test of time. The study presented here is a well-planned, properly formulated, and evidence-based trial that has led to the development of a therapeutic drug that may aid in the “Elimination of Hepatitis by 2030,” a target of the WHO.

Nasal therapeutic vaccine (NASVAC) is prepared by adding two antigens of HBV, as both cellular and humoral antigen-specific immunity of these two antigens is essential for containment of HBV replication and control of liver damage. Additionally, we formulated NASVAC to induce mucosal immunity by producing the nasal form of NASVAC. As antigens administered *via* the nasal route can induce both cytotoxic T cells and CD4-positive T cells and nasal administration (18–20), we opted to use NASVAC for treating CHB patients. In addition, the treatment is finite and ends in 20 weeks.

The communication followed the last four articles that reported phase I/II clinical trials, 24 weeks after EOT of phase III clinical trials, and two and two 3-years after EOT of phase III trial (21–24). The study revealed that most patients with CHB receiving NASVAC exhibited excellent safety profiles in all related fields. Additionally, NASVAC administration was associated with HBV DNA negativity in the sera of a considerable number of patients with CHB. In addition, ALT, a marker of liver damage, was normalized in approximately 66% of patients with CHB 5 years after EOT. None of the patients developed LC or HCC, which was confirmed by abdominal ultrasonography and endoscopic assessment of esophageal varices.

The most notable aspect of this study was related to the route of administration of NASVAC and its finite mode of therapy. Although we used NASVAC 10 times *via* the intranasal and subcutaneous routes in this trial, we have been conducting clinical trials by administrating NASVAC *via* the intranasal route ten times in Japanese patients with CHB (25). The outcome has shown profound therapeutic efficacy of NASVAC in these patients, with a significant reduction in serum HBsAg levels (25).

The intranasal administration of NASVAC has tremendous clinical and social importance. According to the WHO, out of 296 million chronic HBV-infected patients, only 10% are aware of HBV infectivity. The millions of missing HBV-infected patients primarily reside in developing and resource-constrained countries. The treatment of these millions of patients depends on the availability of a finite therapeutic regimen and patient-friendly protocol. Thus, NUCs are unsuitable for millions of patients because there is a need to take them for an infinite duration or even for life. Cessation of NUCs may cause hepatic failure or other severe complications with fatal outcomes. However, NASVAC can be performed by patients themselves if proper training is provided. Thus. NASVAC can alter the overall picture of CHB therapy.

The most common concern regarding nasal administration was addressed by checking all patients by ENT specialists. The second challenge was the mode of administration of NASVAC. NASVAC was performed by tilting the head, and all patients complied with the procedures. We also checked whether self-medication by the intranasal route was possible. It was found that most of the elderly patients were competent to receive NASVAC by their elves after short-term training.

Another pertinent limitation of evolving therapeutics has been resolved by regularly publishing data on NASVAC treatment (prior to therapy, EOT; 24 weeks after EOT; and 2 and 3 years after EOT) (22–24). This is the first report of the publication of follow-up data on any evolving therapy for CHB. This is an extremely important step in the development of new, novel, and evolving therapies. Hundreds of immune therapies for CHB have been reported. Unfortunately, most of the studies have provided data on EOT, and it is elusive as to what happens in long-term follow-up. One or two studies may have provided follow-up data 1 year after EOT. The present study is the first and only study that has attempted to provide long-term follow-up data after EOT (5-year after follow up). This encouraging outcome will provide inspiration for the development of new and novel immune therapeutic approaches for CHB.

One of the major limitations of this study is the lack of assessment of quantitative HBsAg (qHBsAg). In fact, when this study was accomplished in Bangladesh, it was not possible to assess qHBsAg over there. However, it is expected that NASVAC may have a role on qHBsAg levels. A study conducted by us in Ehime University, Japan has shown that NASVAC reduces qHBsAg in their cohort (25). Also, a study is progress in Bangladesh to assess the role of NASVAC on treatment-naïve patients of CHB.

In conclusion, we have an unmasked immune therapeutic, NASVAC, which is safe and highly effective for containing HBV DNA and ALT and blocking progression to LC and HCC. It is a finite therapeutic regimen that can be used quickly *via* the nasal route to stimulate the mucosal immunity. The data for 5-year after EOT indicate that a new nasal drug is knocking on the door, and this may be rationally used to attain the goal of “Elimination of Hepatitis by 2030” in all countries, including developing and resource-constrained countries.

## Data availability statement

The raw data supporting the conclusions of this article will be made available by the authors, without undue reservation.

## Ethics statement

The studies involving human participants were reviewed and approved by Bangabandhu Sheikh Mujib Medical University, Dhaka, Bangladesh, with the project identification code NO. BSMMU/2010/2363, dated March 1, 2010. Written informed consent to participate in this study was provided by the participants’ legal guardian/next of kin.

## Author contributions

MM, SA, and JA: conceptualization, writing—original draft preparation. MM and JA: methodology, validation, investigation. JA, SA, and SK: formal analysis. MM, JA, and SK: resources. JA and SA: data curation. GG, OY, and YH: writing—review and editing. MM: project administration. MM and SA: funding acquisition. All authors contributed to the article and approved the submitted version.

## Funding

This study was supported in part by a grant-in-aid from the Japan Agency for Medical Research and Development (AMED) to SA (grant number 20fk0310103h1905).

## Conflict of interest

The authors declare that the research was conducted in the absence of any commercial or financial relationships that could be construed as a potential conflict of interest.

## Publisher’s note

All claims expressed in this article are solely those of the authors and do not necessarily represent those of their affiliated organizations, or those of the publisher, the editors and the reviewers. Any product that may be evaluated in this article, or claim that may be made by its manufacturer, is not guaranteed or endorsed by the publisher.

## References

[1] WHO. Global Hepatitis Report 2017. Geneva: World Health Organization (2017).

[2] Zhang YY Hu KQ Duan Z. New perspective on the natural course of chronic HBV infection. Front Med. (2014) 8:129–34. doi: 10.1007/s11684-014-0339-x, PMID: 24871442

[3] Chang ML Liaw YF. Hepatitis B flares in chronic hepatitis B: pathogenesis, natural course, and management. J Hepatol. (2014) 61:1407–17. doi: 10.1016/j.jhep.2014.08.033, PMID: 25178562

[4] Ozaras R Khodor H Yetim N Unal UK Demirhan YE Gultekin G. Monotherapy for hepatitis B infection: a review of treatment options. Expert Rev Anti-Infect Ther. (2015) 13:1457–68. doi: 10.1586/14787210.2015.1093934, PMID: 26414781

[5] Terrault NA Bzowej NH Chang KM Hwang JP Jonas MM Murad MH. AASLD guidelines for treatment of chronic hepatitis B. Hepatology. (2016) 63:261–83. doi: 10.1002/hep.28156, PMID: 26566064 PMC5987259

[6] Viganò M Grossi G Loglio A Lampertico P. Treatment of hepatitis B: is there still a role for interferon? Liver Int. (2018) 38:79–83. doi: 10.1111/liv.13635, PMID: 29427498

[7] Viganò M Mangia G Lampertico P. Results of treatment of chronic hepatitis B with pegylated interferon. Clin Liver Dis. (2013) 17:425–43. doi: 10.1016/j.cld.2013.05.004 23905814

[8] Wu J Yin F Zhou X. Efficacy of nucleoside analogues for hepatitis B virus-related liver failure: a network meta-analysis. Acta Pharma. (2018) 68:19–30. doi: 10.2478/acph-2018-0010, PMID: 29453915

[9] Dienstag JL. Benefits and risks of nucleoside analog therapy for hepatitis B. Hepatology. (2009) 49:S112–21. doi: 10.1002/hep.22920 19399795

[10] Liem KS Fung S Wong DK Yim C Noureldin S Chen J. Limited sustained response after stopping nucleos(t)ide analogues in patients with chronic hepatitis B: results from a randomised controlled trial (Toronto STOP study). Gut. (2019) 68:2206–13. doi: 10.1136/gutjnl-2019-318981, PMID: 31462554

[11] Leoni S Casabianca A Biagioni B Serio I. Viral hepatitis: innovations and expectations. World J Gastroenterol. (2022) 28:517–31. doi: 10.3748/wjg.v28.i5.517, PMID: 35316960 PMC8905017

[12] Gill US Kennedy PTF. The impact of currently licensed therapies on viral and immune responses in chronic hepatitis B: considerations for future novel therapeutics. J Viral Hepat. (2019) 26:4–15. doi: 10.1111/jvh.13040, PMID: 30415490

[13] Barili V Vecchi A Rossi M Montali I Tiezzi C Penna A. Unraveling the multifaceted nature of CD8 T cell exhaustion provides the molecular basis for therapeutic T cell reconstitution in chronic hepatitis B and C. Cells. (2021) 10:2563. doi: 10.3390/cells10102563, PMID: 34685543 PMC8533840

[14] Wu J Han M Li J Yang X Yang D. Immunopathogenesis of HBV infection. Adv Exp Med Biol. (2020) 1179:71–107. doi: 10.1007/978-981-13-9151-4_4, PMID: 31741334

[15] Maini MK Boni C Lee CK Larrubia JR Reignat S Ogg GS. The role of virus-specific CD8(+) cells in liver damage and viral control during persistent hepatitis B virus infection. J Exp Med. (2000) 191:1269–80. doi: 10.1084/jem.191.8.1269, PMID: 10770795 PMC2193131

[16] Pol S Driss F Michel ML Nalpas B Berthelot P Brechot C. Specific vaccine therapy in chronic hepatitis B infection. Lancet. (1994) 344:342. doi: 10.1016/s0140-6736(94)91384-6 7914291

[17] Akbar SMF Yoshida O Hiasa Y. Immune therapies against chronic hepatitis B. J Gastroenterol. (2022) 57:517–28. doi: 10.1007/s00535-022-01890-8, PMID: 35708793 PMC9308615

[18] Lobaina Y Palenzuela D García D Rodríguez D Pichardo D Muzio V. Comparative study of the immunogenicity and immunoenhancing effects of two hepatitis B core antigen variants in mice by nasal administration. Vaccine. (2006) 24:S58–9. doi: 10.1016/j.vaccine.2005.01.122, PMID: 16823928

[19] Betancourt AA Delgado CA Estévez ZC Martínez JC Ríos GV Aureoles-Roselló SR. Phase I clinical trial in healthy adults of a nasal vaccine candidate containing recombinant hepatitis B surface and core antigens. Int J Infect Dis. (2007) 11:394–401. doi: 10.1016/j.ijid.2006.09.010, PMID: 17257877

[20] Akbar SM Yoshida O Chen S Cesar AJ Abe M Matsuura B. Immune modulator and antiviral potential of dendritic cells pulsed with both hepatitis B surface antigen and core antigen for treating chronic HBV infection. Antivir Ther. (2010) 15:887–95. doi: 10.3851/IMP1637, PMID: 20834101

[21] Al-Mahtab M Akbar SM Aguilar JC Uddin MH Khan MS Rahman S. Therapeutic potential of a combined hepatitis B virus surface and core antigen vaccine in patients with chronic hepatitis B. Hepatol Int. (2013) 7:981–9. doi: 10.1007/s12072-013-9486-4, PMID: 26202028

[22] Al Mahtab M Akbar SMF Aguilar JC Guillen G Penton E Tuero A. Treatment of chronic hepatitis B naïve patients with a therapeutic vaccine containing HBs and HBc antigens (a randomized, open and treatment-controlled phase III clinical trial). PLoS One. (2018) 13:e0201236. doi: 10.1371/journal.pone.0201236, PMID: 30133478 PMC6104936

[23] Akbar SMF Al Mahtab M Aguilar JC Yoshida O Penton E Gerardo GN. Sustained antiviral and liver protection by a nasal therapeutic vaccine (NASVAC), containing both HBsAg and HBcAg: 2-year follow-up of phase III clinical trial. Pathogens. (2021) 10:1440. doi: 10.3390/pathogens10111440, PMID: 34832596 PMC8619282

[24] Akbar SMF Al Mahtab M Aguilar JC Yoshida O Khan S Penton E. The safety and efficacy of a therapeutic vaccine for chronic hepatitis B: a follow-up study of phase III clinical trial. Vaccines (Basel). (2021) 10:45. doi: 10.3390/vaccines10010045, PMID: 35062707 PMC8778341

[25] Yoshida O Akbar SMF Imai Y Sanada T Tsukiyama-Kohara K Miyazaki T. Intranasal therapeutic vaccine containing HBsAg and HBcAg for patients with chronic hepatitis B; 18 months follow-up results of phase IIa clinical study. Hepatol Res. (2022). doi: 10.1111/hepr.13851, PMID: 36399406

